# Bone Marrow Adipocytes, Adipocytokines, and Breast Cancer Cells: Novel Implications in Bone Metastasis of Breast Cancer

**DOI:** 10.3389/fonc.2020.561595

**Published:** 2020-10-02

**Authors:** Chang Liu, Qian Zhao, Xijie Yu

**Affiliations:** ^1^Department of Endocrinology and Metabolism, Laboratory of Endocrinology and Metabolism, National Clinical Research Center for Geriatrics, West China Hospital, Sichuan University, Chengdu, China; ^2^Department of General Practice, West China Hospital, Sichuan University, Chengdu, China

**Keywords:** bone marrow adipocyte, breast cancer, bone metastasis, adipocytokine, adipokine

## Abstract

Accumulating discoveries highlight the importance of interaction between marrow stromal cells and cancer cells for bone metastasis. Bone is the most common metastatic site of breast cancer and bone marrow adipocytes (BMAs) are the most abundant component of the bone marrow microenvironment. BMAs are unique in their origin and location, and recently they are found to serve as an endocrine organ that secretes adipokines, cytokines, chemokines, and growth factors. It is reasonable to speculate that BMAs contribute to the modification of bone metastatic microenvironment and affecting metastatic breast cancer cells in the bone marrow. Indeed, BMAs may participate in bone metastasis of breast cancer through regulation of recruitment, invasion, survival, colonization, proliferation, angiogenesis, and immune modulation by their production of various adipocytokines. In this review, we provide an overview of research progress, focusing on adipocytokines secreted by BMAs and their potential roles for bone metastasis of breast cancer, and investigating the mechanisms mediating the interaction between BMAs and metastatic breast cancer cells. Based on current findings, BMAs may function as a pivotal modulator of bone metastasis of breast cancer, therefore targeting BMAs combined with conventional treatment programs might present a promising therapeutic option.

## Introduction

Breast cancer is the most common cancer among women and it leads to the second most tumor-related deaths in women worldwide (1, 2). Great progress in the development of better diagnosis and treatment for this cancer has been achieved and contributes significantly to the decline in the mortality rate. However, breast cancer still accounts for more than a half-million deaths worldwide annually (3). This high mortality rate is mainly on account of the difficulty to cure metastatic disease. Bone is the most common metastatic site in advanced breast cancer (4). Bone metastasis drastically impacts the quality of life and survival of breast cancer patients (5). Therefore, it is essential to explore the mechanism of bone metastasis of breast cancer.

Due to the unfamiliar environment at the secondary site, the metastatic process is described to be inefficient, compared to tumor development at the primary site. Actually, only a small group of disseminated tumor cells (DTCs) initiate metastatic growth (6). Upon arrival to the bone, bone marrow may offer an ideal soil for DTCs (seeds). The bone marrow microenvironment comprises of multiple cell types, such as osteoblasts, osteoclasts, hematopoietic cells, mesenchymal stem cells, endothelial cells, and adipocytes. All of these cells play indispensable roles in the maintenance of bone homeostasis (7). Furthermore, they might provide a supportive niche for metastatic cancer cells. Many efforts have been made to uncover the functions of the bone marrow microenvironment and research the role of each cell type in tumor growth and metastasis (8).

The contribution of stromal cells including osteoclasts, osteoblasts, and inflammatory cells to bone metastasis of breast cancer has been extensively described (7). Occupying the highest proportion of the bone marrow, however, the comprehensive roles of bone marrow adipocytes (BMAs) in the metastatic microenvironment are still poorly understood (9). BMAs are the most abundant component of stromal cells in the bone marrow niche. They progressively increase with aging (10). In children, 15% of bone marrow is composed of adipocytes approximately, while in adolescent, adipocytes occupy 70% volume of long bone marrow (11).

At present it is widely accepted that there are at least three types of adipocytes: white, brown, and beige. This classification is based on their appearance, function, and site of origin (12). Although BMAs possess some characteristics of white adipocytes, they appear to be a distinct fourth population of adipocytes, a previously unrecognized fat depot (13). BMA is characterized by a unilocular lipid droplet within a cytoplasm that is surrounded by a lipid membrane and an adjacent single nucleus. Although it is often argued that BMA has beige characteristics because of modest Ucp1 gene expression in some animal models, no researcher has definitively shown thermogenic capability in bone marrow adipose tissue, nor significant protein expression of UCP1 (12). The unique phenotype of BMAs is confirmed by comparison of gene markers characteristic to white, brown, and beige adipocytes. BMAs do not express white-exclusive Tcf21 marker, brown-exclusive Zic1 marker, and beige-specific Tmem26 marker, suggesting their different phenotype from peripheral white, brown, and beige adipocytes (14).

Based on a very recent research, though their origins are different, BMAs and white adipocytes (including abdominal, visceral, and subcutaneous adipocytes) have many common characteristics (15). These two types of adipocytes are not only similar in morphology, but also present similar protein secretion profiles. The cytokines expressed by BMAs are also expressed in white adipocytes. The effects of BMAs-derived cytokines on breast cancer is the same as that of white adipocytes-derived cytokines. Therefore, the roles of BMAs on breast cancer is similar to that of white adipocytes.

For a long time, BMAs have been described to fill the interspace of the bone marrow. Nevertheless, recently BMAs are demonstrated to function as an endocrine organ (7). BMAs can secrete various bioactive peptides or proteins. These molecules are named as adipocytokines collectively. The terms adipokine and adipocytokine are usually used synonymously. Accurately, adipocytokines refer to all factors secreted by adipocytes, including adipokines, cytokines, chemokines, and growth factors. Adipokines are some factors that are secreted mainly but not exclusively by adipocytes (16).

So far, BMAs have been demonstrated to release adipokines such as leptin and adiponectin (11); cytokines such as interleukin-6 (IL-6) (11), IL-1β (11), tumor necrosis factor-α (TNF-α) (11), receptor activator of nuclear factor kappa-B ligand (RANKL) (12, 17); chemokines such as chemokine (C-X-C motif) ligand 1 (CXCL1) (11), CXCL2 (11), CXCL5 (18), CXCL12 (10), C-X3-C motif ligand 1 (CX3CL1) (19, 20), C-C motif ligand 2 (CCL2) (21, 22); and growth factors such as insulin-like growth factor-1 (IGF-1) (10), fibroblast growth factor-2 (FGF-2) (10). Recently, a few novel adipokines including angiopoietin-like protein 2/4 (ANGPTL2/4) (10, 23), chemerin (24), fatty acid-binding protein 4 (FABP4) (10, 23), lipocalin 2 (LCN2) (25, 26), resistin (23) and visfatin (10, 23) are found to be produced by BMAs. Via these adipocytokines, BMAs influence the cells in the bone marrow by autocrine, endocrine, and paracrine signaling. However, at present no adipocytokines expressed specifically by BMAs has been found.

BMAs are the most abundant component in the bone marrow microenvironment, especially among postmenopausal women (12). Interestingly, postmenopausal women are the population with a high incidence of bone metastasis of breast cancer. The effect of BMAs on local tumor cells in bone marrow may be greater than other marrow stromal cells such as mesenchymal stem cells, endothelial cells, and fibroblasts.

Increasing evidence has highlighted the important role of adipocytokines as an active player involved in breast cancer progression and metastasis by remodeling extracellular matrix (ECM), modulating immune responses, influencing epithelial-mesenchymal transition (EMT), inducing cancer stem cell-like traits, increasing cancer cells proliferation and growth, and regulating angiogenesis (27). In this review, we provide an overview of research progress, focusing on secreted adipocytokines by BMAs and their potential roles for bone metastasis of breast cancer, and investigating the mechanisms mediating the interaction between BMAs and metastatic breast cancer cells. Several novel adipokines are especially emphasized as new evidence is emerging regarding their involvement in bone metastasis of breast cancer.

## BMAs and Mechanisms Involved in Pre-Metastatic Niche Formation

**Figure 1:**
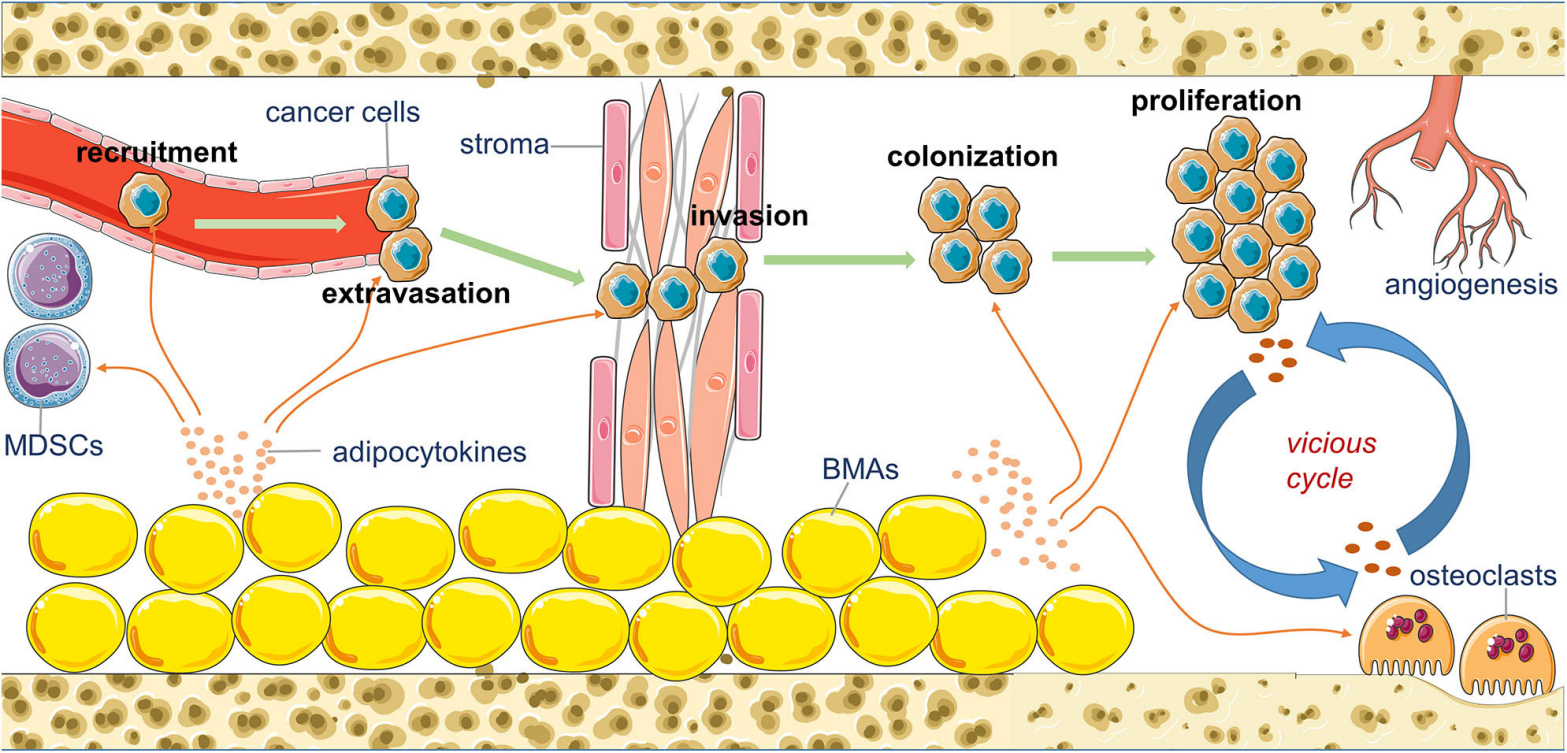
An overview of the potential contribution of bone marrow adipocytes (BMAs) to the bone metastasis of breast cancer. BMAs affect the recruitment, extravasation, invasion, colonization, proliferation, and angiogenesis of metastatic breast cancer cells in the bone marrow by their secreting various adipocytokines.

The formation of bone metastasis is a multi-step process. It includes attraction of chemoattractants to circulating tumor cells (CTCs), departure of cancer cells from blood vessels (extravasation), local invasion and migration, colonization and adaption, and expanded growth to macrometastasis. Each step demands close cooperation of cancer cells with the specific partners in the bone microenvironment (20). The remaining section of this review elaborates on the acknowledged functions of adipocytokines in the adipocyte-breast cancer cell interaction and the potential role that BMA-secreted adipocytokines may play in bone metastasis of breast cancer during each stage (Figure 1).

Increasing discoveries reveal that tumors lead to the development of an appropriate microenvironment in secondary organs that conduce to the colonization and growth of CTCs before they arrive at these sites (28). This predetermined microenvironment is termed “pre-metastatic niche” (PMN). Various studies have identified some mechanisms that regulate complicated molecular and cellular changes in the PMN to support the next growth of metastatic tumors (29).

Bone is a frequent metastatic site for some types of solid tumors, such as breast, prostate, and lung cancer. BMAs represent the major population of bone marrow cells (13). BMAs and the BMAs-secreted factors can impact some resident cells and matrix in the bone marrow to develop a PMN and the next colonization of metastatic cells (30). Concretely, the steps of PMN formation consist of the promotion of vascular leakiness, the remodeling of ECM, and immune modulation (31).

### Adipocytokines and Vessel Barrier Breakdown: Permeability

The vasculature at PMN is remodeled by adipocyte-secreted adipocytokines in various ways. Animal tumor models indicate promoted vascular permeability at PMN (32), which is related to increased extravasation and metastatic burden. Adipocytokines, such as the inflammatory cytokines IL-6, IL-1β, TNF-α can induce vascular permeability in bone marrow, enabling CTCs to extravasate. As we know, extravasation is the initiating step of bone metastasis.

Novel adipokines also contribute to this process. ANGPTL2 and ANGPTL4 enhance the permeability of microvessels in the premetastatic sites synergistically (33). Visfatin, another adipokine, activates Nlrp3 inflammasome to remarkably decrease the expression of inter-endothelial junction proteins, including tight junction proteins ZO-1, ZO-2, occludin, and adherens junction protein vascular endothelial (VE)-cadherin. These disrupt inter-endothelial junctions and increase paracellular permeability of the endothelium (34).

Chemokine CXCL12 also enhances tumor vasculature permeability, facilitating colonization to distant organs. Mechanistically, CXCL12 acts on endothelial cells through its receptor chemokine (C-X-C motif) receptor 4 (CXCR4) and promotes trans-endothelial migration of tumor cells. Analogous to visfatin, CXCL12 inhibits the expression of junction proteins including ZO-1, occludin, and VE-cadherin (35).

Consequently, metastatic osteotropism likely requires specific interactions of BMAs with endothelial cells in the bone marrow. Metastasis prefers to direct to bone marrow because the bone marrow vasculature is relatively permissive for tumor cell extravasation (29).

### Adipocytokines and Remodeling of the ECM

The ECM at PMN undergoes significant changes. The lysyl oxidases (LOX) family induce the crosslinking of ECM, which plays an important role in ECM-shaping (30). LOX exerts its pro-metastatic effect via enhancing the stiffness of the ECM to promote the anchorage and colonization of cancer cells (36, 37).

It has been demonstrated that the ECM modifying enzyme LOX secreted by breast cancer cells induces a metastatic niche in bone. This forms a PMN that can act as a platform for CTCs to colonize (38). Adipocytokines such as TNF-α, IL-1β, FGF-2, and CCL2, have been found to increase LOX expression in breast cancer (29, 39, 40). For example, TNF-α induces LOX expression via the reactive oxygen species-activated nuclear factor-kappaB (NF-κB)/extracellular signal-related kinase (ERK) pathway, thus promoting the progression of breast cancer metastasis (40). Collectively, collagen crosslinking induced by adipocytokines leads to enhanced tissue stiffness to facilitate tumor cell seeding and accelerate metastatic growth (29).

### Adipocytokines and Immune Modulation

Anti-tumor immunity mediated by immune cells such as natural killer (NK) cells and T cells that attack tumor cells is a natural defense against cancer. To overcome this barrier, immunosuppressive mechanisms at metastatic sites recruit other immune cells that can suppress these anti-tumor responses. This recruitment of immune cells is a hallmark of PMN establishment (41). Accumulation of immunosuppressive myeloid cell populations can limit anti-tumor adaptive immunity to promote metastatic spread. These immunosuppressive myeloid cell populations are collectively termed myeloid derived suppressor cells (MDSCs) (42). In the bone marrow, MDSCs can be recruited by BMAs-derived adipocytokines to form a tumor-favoring microenvironment to suppress the anti-tumor immune response (43).

Several proinflammatory cytokines, such as IL-6, IL-1β, TNF-α, facilitate the cancer progression by recruiting and activating MDSCs at the sites of the next metastases. Members of the signal transducer and activator of transcription (STAT) family play important roles in promoting the differentiation of MDSCs stimulated by these cytokines (44, 45).

An HFD and the accompanying obesity induce the accumulation of excess numbers of MDSCs in mice. This result enhances spontaneous metastasis. Mechanistically, the induction of MDSC is regulated by leptin, a classic adipokine that is overexpressed in HFD mice and the accumulation of MDSCs can be reduced by blocking the leptin receptor (45).

Some chemokines and chemokine receptors also play roles in recruiting MDSCs in PMN. In different types of cancers (including breast cancer and melanoma), CXCL1, CXCL2, and CXCL5 with their common receptor CXCR2 trigger the recruitment of MDSCs into the PMN (46). Moreover, the CXCL12/CXCR4 signaling pathway promotes MDSCs trafficking in the tumor microenvironment (47). CCL2 is implicated in the recruitment of MDSCs in several murine cancer models, including lung carcinoma, melanoma, colorectal cancer, and breast cancer. Interestingly, its receptor CCR2 deficiency results in a significant decrease in colon cancer growth through the inhibition of MDSCs infiltration (48).

## BMAs and Mechanisms that Participate in the Recruitment of Cancer Cells to the Bone Marrow

Paget's seed and soil hypothesis postulates that the specific organ microenvironment recruits and supports the survival and growth of specific types of cancer cells. The osteotropism feature of breast cancer suggests the presence of specific factors from the bone that activate the recruitment of breast cancer cells to the bone marrow (49).

To date, some types of bone marrow cells have been evaluated for their contribution to attracting breast cancer cells, such as osteoblasts, osteoclasts, and adipocytes (50, 51). These studies report that some adipocytokines are involved in breast cancer osteotropism, including CXCL12, RANKL, leptin, and IL-1β.

### Chemokines and Breast Cancer Osteotropism

It is well-documented that some chemokines contribute to the recruitment of CTCs to the bone marrow. The BMAs-secreted chemokines can establish a concentration gradient between the bone marrow and the blood circulation, which guides the cancer cells homing to bone marrow. CXCL12 is reported to be released in the bone marrow. The breast-derived CTCs expressing CXCL12 receptor prefer to move to the bone marrow because of the chemotactic ability of CXCL12. On the contrary, treatment with an antibody of the CXCL12 receptor partly decreases the formation of bone metastases of prostate cancer (37).

Meanwhile, other adipocytokines assist this process. ANGPTL2 and IL-6 enhance the reaction of breast cancer cells to bone-derived CXCL12 through the upregulation of CXCL12 receptor CXCR4 in these cancer cells, respectively (52, 53). Additionally, the CXCL12/CXCR4 axis can increase the expression of cell-endothelium adhesion molecules in the bone marrow, such as vascular cell adhesion molecule-1 (VCAM1) and integrins α4β1, which further enhances multiple myeloma cells homing to the marrow (54). Interestingly, the phenomenon that expression of CXCR4 facilitates cells to move to the CXCL12-expressed bone marrow has been found in other types of tumors, such as prostate and lung cancer (20).

CXCL10 is another chemokine produced in the BMAs that motivates the directional movement of breast cancer cells by its receptor CXCR3 on cancer cells (10). Accordingly, treatment with an antibody of CXCL10 reduces bone marrow colonization of breast cancer cells (37).

Moreover, chemokine CX3CL1 has been demonstrated to mediate specific metastasis of breast cancer to the bone marrow. Breast cancer cells that express CX3CL1 receptor CX3CR1 show a high preference for metastasizing to the bone. Metastasis to the bone is decreased in CX3CL1 knockout mice, while it does not impact metastasis to adrenal glands. This result means that CX3CL1 is necessary for breast CTCs to home to bone marrow (20).

### Cytokines and Breast Cancer Osteotropism

The RANKL can be secreted as a cytokine by BMAs. Interestingly, RANK, the RANKL receptor, is also expressed by breast cancer cells. RANKL can promote the movement of cancer cells (breast or prostate cancer) that express RANK (37). Therefore, RANKL may involve the recruitment of cancer cells to the bone marrow. In turn, treatment with osteoprotegerin (OPG), an inhibitor of the RANK/RANKL axis, inhibits the development of bone metastases of breast cancer (55).

### Adipokines and Breast Cancer Osteotropism

In a human bone tissue explant model, when breast cancer cells are co-cultured with bone tissue fragments, cancer cells are inclined to migrate toward the BMAs. Mechanism research reveals that breast cancer cells are recruited to the human bone tissue by leptin and IL-1β derived from BMAs (49). This study demonstrates a direct effect of BMAs on breast cancer.

## BMAs and Mechanisms Associated With Cancer Cells Migration and Invasion in the Bone Marrow

Accumulating evidence suggests that EMT and cancer stem cell (CSC) characteristics involve tumor progression, both of which contribute to tumor migration, invasion, and metastasis. Furthermore, both of them can be correlated with BMAs-secreted adipocytokines (56). In the EMT process, epithelial tumor cells obtain a mesenchymal phenotype to enhance cell motility and invasiveness (57). EMT has an important role in cancer invasion and metastasis. It is also a mechanism involved in bone metastasis formation (58).

**Figure 2:**
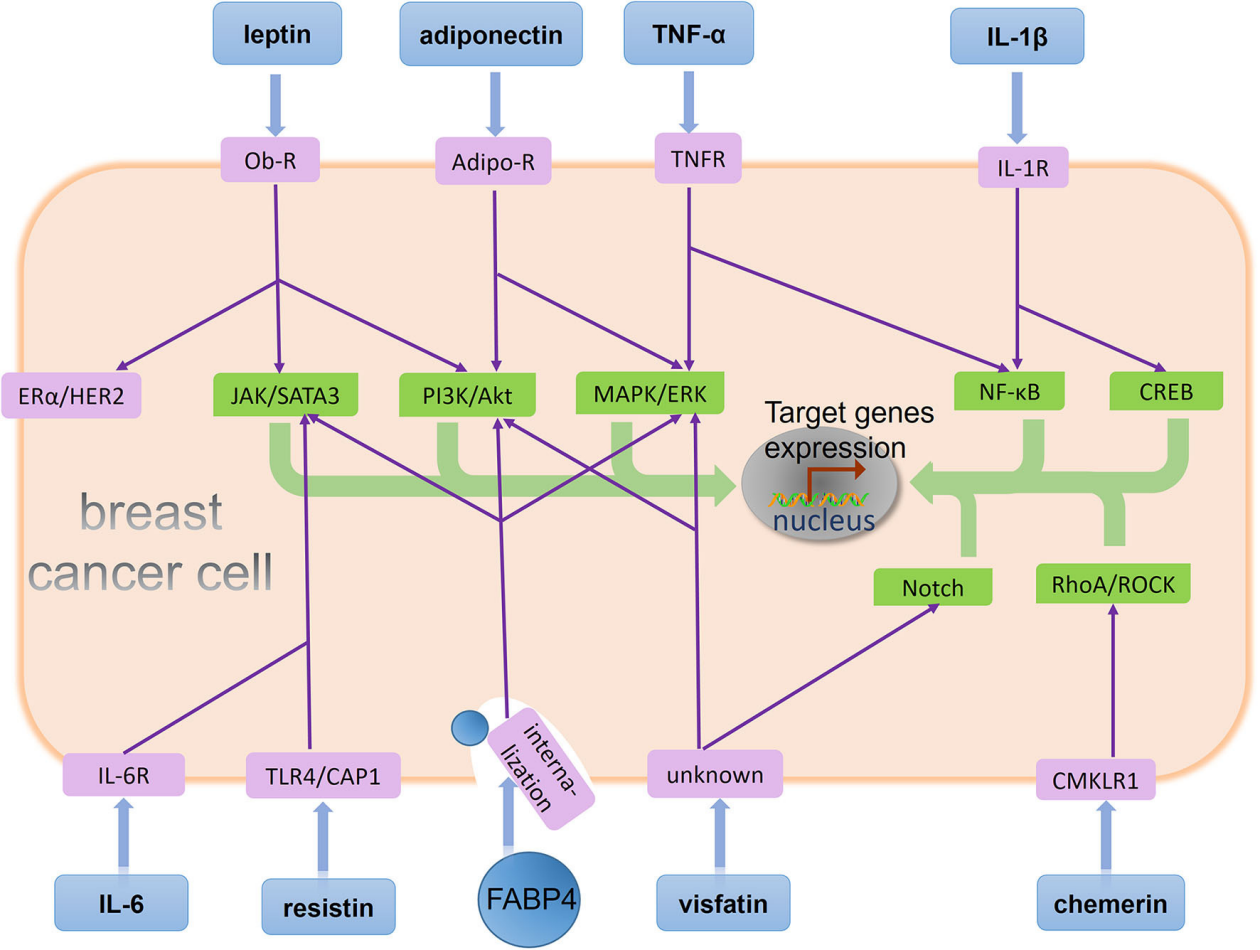
BMAs-derived adipocytokines regulate behavior of metastatic breast cancer cells in the bone marrow. A few adipocytokines act on their corresponding receptors on breast cancer cell and affect downstream signaling pathways. Specifically, leptin binds its receptor on the breast cancer cell, Ob-R, and stimulates the JAK/SATA3 and PI3K/Akt signaling pathway. Moreover, leptin has activation effects in ERα and HER2 independent of their ligands. Adiponectin is recognized by its receptor Adipo-R on the breast cancer cell, and two signaling pathway PI3K/Akt and MAPK/ERK are regulated by adiponectin. TNF-α induces signaling cascades in cancer cells mediated by its receptor TNFR, including MAPK/ERK and NF-κB activation. IL-1β upregulates NF-κB and CREB activation via its receptor IL-1R. IL-6 binds its receptor IL-6R, and resistin binds its receptor TLR4 or CAP1. Both of them stimulates the JAK/SATA3 signaling pathway. FABP4 enhances three different signaling pathway: JAK/SATA3, PI3K/Akt, and MAPK/ERK after its internalization by breast cancer cell. Visfatin binds an unknown receptor on the breast cancer cell, and stimulates the MAPK/ERK and Notch signaling pathway. Chemerin upregulates RhoA/ROCK activation via its receptor CMKLR1. Eventually, these adipocytokines stimulate different signaling pathways including JAK/SATA3, PI3K/Akt, MAPK/ERK, NF-κB, CREB, Notch, RhoA/ROCK, ERα, and HER2 to promote target genes expression and regulate different tumor biological processes such as proliferation, EMT, stemness, and angiogenesis.

In addition, EMT contributes to the development and maintenance of breast CSCs (59), a cell population possessing the property for self-renewal and initiating a secondary cancer. Preclinical studies demonstrate that stem-like phenotypes are responsible for bone metastases of breast cancer (57). Clinical studies show the presence of cancer cells with CSCs capabilities in the bone marrow of breast cancer patients (58). Therefore, it is necessary to identify adipocytokines that stimulate the development of EMT and CSCs (Figure 2).

### Leptin and EMT

Previous studies have shown that leptin promotes EMT via many mechanisms (27). For instance, there is a potential cross-talk between leptin and metastasis-associated protein 1 (MTA1)/Wnt signaling in EMT of breast cancer cell lines (60). Leptin-induced IL-8 activation via intracellular signaling molecules, such as STAT3, Akt, and ERK 1/2, facilitates EMT of breast cancer cells (61). The treatment of breast cancer MCF-7 cell line with leptin leads to a remarkable increase in the expression of EMT markers (including vimentin and Snail) along with a downregulation of the epithelial marker E-cadherin (62). Besides, leptin secreted by adipose stem cells is demonstrated to promote the mesenchymal phenotype in triple-negative breast cancer (TNBC) cells through increased expression of TWIST1, Serpine1, and SNAI2 (63). Mouse mammary tumor virus (MMTV)-Wnt-1 transgenic mice, which develop spontaneous breast cancer under a diet-induced obesity regimen, present increased leptin production, upregulated EMT gene expression, and reduced survival (64).

### Leptin and CSC

The first proof of this adipokine involved in breast CSC enrichment is from reduced CSC potential of residual tumors from leptin-deficient mice, compared to those from wild-type mice (65). In subsequent studies, activation of ObR (leptin receptor) signaling is reported to be essential for maintaining CSC-like and metastatic properties in TNBC (66). Interestingly, HFD mice with a high leptin level also show upregulated CSC gene expression and enhanced tumoral aldehyde dehydrogenase (ALDH) enzymatic activity, a well-known CSC marker (64). Furthermore, leptin can recruit G9a histone methyltransferase through STAT3 signaling activation. This causes repression of miR-200c, which accelerates the formation of breast CSCs (67). Besides, a leptin/JAK/STAT3-dependent fatty acid β-oxidation signaling is identified to be critical for breast CSC formation. Accordingly, targeting this fatty acid β-oxidation pathway inhibits leptin-induced breast cancer stemness (68). Therefore, leptin, acting as a mediator of the interaction between cancer cells and adipocytes, impacts breast CSC activity (27).

### IL-1β and EMT, CSC

A research using the MCF-7 cells suggests that IL-1β promotes migration and invasion via a mesenchymal phenotype (69). A non-canonical activation of IL-1β-mediated β-catenin signaling is reported to lead to the onset of EMT in breast cancer cells (70). The induction of EMT in breast cancer by IL-1β also links to an NF-κB-dependent mechanism (71). In a humanized model of spontaneous breast cancer metastasis to bone, production of IL-1β by cancer cells promotes EMT (altered E-Cadherin, N-Cadherin, and G-Catenin), invasion, migration, and bone colonization. Inhibitor of IL-1β, Anakinra or Canakinumab, reduces metastasis and the number of cancer cells shed into the circulation (72). Clinical data show that continuous inhibition of IL-1 activity inhibits breast cancer growth and bone metastasis (73).

In the bone metastatic niche, microenvironmental IL-1β enhances the ability of breast CSCs to form colonies by activation of NF-κB and cAMP-response element-binding protein (CREB) signaling, Wnt ligand secretion, and autocrine Wnt signaling in breast cancer cells. Besides, blockage of this IL-1β pathway inhibits both bone metastasis of breast cancer and CSC colony development in the bone environment (74). Collectively, present results demonstrate a functional role of IL-1β signaling in migration and invasion of breast cancer (73).

### IL-6 and EMT

Previous researches have reported that exogenous and endogenous IL-6 can promote breast cancer invasion and migration through the activation of EMT. The mature adipocytes facilitate the invasive behavior of breast cancer cells and trigger an EMT-phenotype via paracrine IL-6/STAT3 signaling (75). In a study of breast cancer T47D cells, IL-6 promotes EMT through the increased activation of ERK1/2 and the phosphorylation of Shp2, a protein tyrosine phosphatase (76). Moreover, there is a direct interplay between the oncoprotein Y-box binding protein-1 (YB-1) and IL-6, which affects breast cancer metastasis. Overexpression of YB-1 in breast cancer induces IL-6 secretion, in turn, treatment with IL-6 increases YB-1 expression, both of which upregulate EMT. This finding reveals a positive feed-forward loop driving EMT-like character between IL-6 and YB-1 (77). A blockade of IL-6 pathway by treatment with niclosamide, metformin, or IL-6 shRNA reverses adipocyte-induced EMT via blocking of IL-6/STAT3 signaling and downregulation of EMT-transcription factors, such as NF-κB, TWIST, and SNAIL, as well as EMT marker vimentin and N-cadherin (78–80).

### IL-6 and CSC

In the exploration of the origins of breast CSCs and their relationships to non-stem cancer cells (NSCCs), a critical role for IL-6 has been found in controlling the dynamic balance between breast CSCs and NSCCs. In a mixed population, NSCCs can be converted to CSCs in response to exogenous or CSC-secreted IL-6 (81). Mechanistically, IL-6 regulates breast CSC-associated OCT4 gene expression through the JAK/STAT3 signal pathway in NSCCs. Inhibiting this pathway by treatment with anti-IL-6 antibody effectively prevents OCT4 gene expression. These results suggest that the IL-6/JAK/STAT3 signal pathway plays an important role in the conversion of NSCCs into CSCs through regulating OCT4 gene expression (82). Besides, IL-6 upregulates Notch-Jagged signaling to expand the proportion of CSCs. In basal-like breast cancer, Notch, Jagged, and IL-6 receptor are overexpressed relative to other breast cancer subtypes. IL-6 promotes JAG1 expression and enhances interaction among cells via Notch3 and JAG1. In turn, Notch3 can facilitate the autocrine production of IL-6. Therefore, the IL-6/Notch3/JAG1 axis sustains mammosphere growth, a feature of breast CSCs (83). In contrast, blocking IL-6 activity reduces breast CSCs formation (84). Esculentoside-A inhibits breast CSCs growth by blocking the IL-6/STAT3 signaling pathway. IL-6/STAT3 pathway proteins including IL-6, phosphorylated STAT3, and STAT3 are downregulated significantly in Esculentoside-A-treated breast CSCs. The expressions of stemness proteins including ALDH1, SOX2, and OCT4 are also reduced. These cause inhibition of proliferation and mammosphere formation of breast CSCs, induce breast CSCs apoptosis, and suppress the cancer growth generated from breast CSCs significantly (85).

### Novel Adipokines and EMT, CSC

FABP4 promotes EMT of breast cancer via the activation of the Akt/GSK3β/Snail pathway (86). It also enhances breast cancer stemness and aggressiveness through stimulating the STAT3/ALDH1 signal (87). LCN2 plays a role in promoting cell migration and invasion of MCF-7 breast cancer cells by inducing EMT (88). Researchers using the MCF-7 cell line discover that resistin facilitates the metastatic potential by the promotion of EMT and stemness, and these effects are primarily attributed to adenylyl cyclase–associated protein 1 (CAP1) (89, 90). Furthermore, resistin is found to promote EMT and CSC-like properties in breast cancer cells through a TLR4/NF-κB/STAT3 signaling pathway (91). Resistin also accelerates invasion and migration of breast cancer cells via stimulating ezrin, radixin, and moesin (ERM) complex, then activated ERM upregulates expression of vimentin, an EMT marker (92). Visfatin induces EMT in mammary epithelial cells by activating the transforming growth factor (TGF) signaling pathway to increase TGF-β1 production (93).

## BMAs and Mechanisms Associated With the Adaptation and Survival of Metastatic Cells in the Bone Microenvironment

It has been postulated that tumor cells migrating to the bone marrow and located in the PMN must adjust to the bone microenvironment for the subsequent formation of overt metastasis (94). To survive in the bone microenvironment, bone metastatic cancer cells attempt to resemble a type of resident bone cells, i.e., the osteoblasts (95). This process, known as osteomimicry, enables tumor cells to survive in the bone marrow microenvironment (37).

Breast cancer cells can undergo osteomimicry after EMT and express factors that are the main mediators of bone remodeling typically found in osteoblasts (95). An osteomimicry profile is characterized by an increased expression of bone sialoprotein (BSP), osteopontin (OPN), osteoprotegerin (OPG), osteonectin (ON), cadherin 11 (CDH11), transcription factor runt-related transcription factor 2 (Runx2) (96), etc. These bone-related genes (BRGs) are highly expressed in bone metastatic cancer cells, compared to those cells metastasized in other organs, and their expression is regulated by the transcription factor Runx2 that acts as a master mediator (97). BMAs-secreted adipocytokines can participate in inducing osteomimicry of breast cancer cells.

### Adipocytokines and Runx2 Signaling Pathway in Osteomimicry

CXCL1 can promote breast cancer migration and invasion ability, as well as EMT in both mouse and human breast cancer cells (98). After CXCL1 treatment, SOX4 expression significantly increases in the nucleus of various breast cancer cell lines (98). SOX4 positively regulates the endothelin-1 expression and facilitates endothelin-1 secretion in breast cancer (99). Endothelin-1 can activate Runx2 and confer an osteomimetic phenotype in breast cancer cells, contributing to colonization and osteolysis (100). Therefore, Runx2 is critical for the CXCL1-induced osteomimetic phenotype by activating the transcription of BRGs in breast cancer cells.

### Adipocytokines and Wnt Signaling Pathway in Osteomimicry

In addition to Runx2, the Wnt/β-catenin pathway also plays an important role in osteoblast differentiation. Interestingly, the Wnt/β-catenin pathway is significantly more expressed in bone metastasis samples of prostate cancer patients (97).

The present studies indicate that leptin and CXCL12 may upregulate the Wnt/β-catenin pathway in breast cancer (101, 102). The miR-218 is an inducer of osteogenesis via activating Wnt signaling. Besides, a positive feedback loop is demonstrated between miR-218 and Wnt signaling (103). Furthermore, highly expressed miR-218 is found in metastatic breast cancer cells compared to normal mammary cells, which increases OPN, BSP, and CXCR4 expression to facilitate tumor growth in the bone (97). Hence, the leptin and CXCL12 activated miR-218/Wnt loop fuels Wnt signaling to enhance expression of metastatic and osteomimetic genes in aggressive breast cancer cells that home to bone (103). Collectively, epithelial breast cancer cells with ectopic expression of BRGs induced by adipocytokines acquire the advantages of residing in the bone microenvironment.

## BMAs and Mechanisms Responsible for Macrometastasis and Outgrowth of Metastasized Cells

Extravasated breast cancer cells need to adapt to specific conditions in the foreign microenvironment to form micrometastases (27). After the development of clinically undetectable micrometastases, breast cancer cells have to grow to form macroscopic metastases. However, metastatic cancer cell proliferation does not occur immediately with a specific temporal pattern because cancer cells seeding at distant bones may remain quiescent until stimulus signals from the bone marrow microenvironment drive cancer cells proliferation into overt metastases in the bone (27). It is found that activated osteoclasts and increased osteoclastic bone resorption accelerate the growth of DTCs into overt metastases (72). In addition, it is hypothesized that when metastatic tumor cells arrive in the bone, they may be stimulated to form overt metastasis through an expansion of the tumor associated vasculature (72). In brief, the process of micrometastatic to macrometastatic transition is involved in cancer cell proliferation, osteoclasts vitality and their bone resorption, as well as angiogenesis. BMAs-derived adipocytokines can play an acceerative role in this process.

### Adipocytokines Associated With Cancer Cells Growth and Proliferation in the Bone Marrow

Adipocytokines not only are associated with the establishment of a pro-tumor microenvironment and organ-directed metastasis but also mediate disease progression, favoring the growth and proliferation of tumor cells (104). Several adipocytokines have been described to participate in these processes (Figure 2).

#### Leptin

Without an estrogen ligand, leptin can activate the estrogen receptor (ER) signaling resulting in the growth of breast cancer cells (105). Several signaling pathways have been demonstrated to enhance proliferative of breast cancer cells, including the activation of JAK/STAT3 and PI3K/Akt by leptin, as well as JAK2 activation-mediated human epidermal growth factor receptor-2 (HER2) transactivation (106, 107). In addition, leptin influences the cell cycle. Leptin upregulates the expression of cyclin D1 and cyclin-dependent kinase 2 (CDK2) but downregulates the expression of p21, p27, and p53, resulting in cell cycle alteration in breast cancer (108).

#### Adiponectin

Adiponectin is reported to inhibit breast cancer growth. However, its effect may depend on the hormonal receptor status (109). In ER-negative breast cancer cells, it reduces cell growth and proliferation (110). Whereas, its effects on ER-positive breast cancer cells are contradictory (111). In ER-positive breast cancer cells, certain concentration adiponectin enables the interaction of APPL1 with adiponectin receptor AdipoR1, ERα, insulin-like growth factor I receptor, and c-Src. This complex stimulates mitogen-activated protein kinase (MAPK) signaling to accelerate breast cancer growth (112). Besides, adiponectin presents different impacts on the cell cycle according to ER status (113). Adiponectin downregulates cyclin in ERα-negative cells and upregulates cyclin in ERα-positive cells, respectively (90).

#### TNF-α

The effects of TNF-α exposure on breast cancer cell lines remain rather contradictory (59). In ER-positive breast cancer cells, TNF-α can promote the proliferation in T47D cells (114), but it presents a pro-apoptotic and anti-mitogenic function in MCF-7 cells (115). In different ER-negative cell lines, TNF-α shows dual effects once more. It accelerates apoptosis in some cases (BT549 cells) (116), however, it enhances survival and proliferation in other cases (MDA-MB-468, SK-BR3, and MDA-MB-231 cells) (117). Therefore, further studies are required to elucidate the role of TNF-a in growth and proliferation.

#### Novel Adipokines

Chemerin increases RhoA/ROCK pathway signal transduction to promote breast cancer cell proliferation and metastasis (118). FABP4 also accelerates cancer cell proliferation by activation of phosphoinositide 3-kinase (PI3K)/Akt and MAPK/ERK pathways and the induction of FOXM1 transcription factor expression in MCF-7 cells (119). Iron facilitates cancer cell proliferation and metastasis. Breast cancer cells show an increased uptake and intracellular storage of iron to support their enhanced metabolism and DNA synthesis (120, 121). Recent evidence supports the existence of transferrin-independent iron transport mechanisms in the tumor microenvironment, which points to local iron transport proteins such as LCN2 (122). Stimulation of breast cancer cells with resistin not only enhances their growth and stemness but also results in chemoresistance through STAT3 activation (123). Visfatin is identified to facilitate the survival and proliferation of breast cancer cells via upregulating Notch1 (124). Visfatin also induces breast cancer cell proliferation and viability through PI3K/Akt and MAPK/ERK activation and protects against apoptosis in these cells (125, 126). Visfatin increases both extracellular and intracellular nicotinamide adenine dinucleotide (NAD) concentration in breast cancer cells, which causes upregulation of silent information regulator 1 (SIRT1) activity and p53 deacetylation. SIRT1 is implicated in blocking senescence and apoptosis and promoting cancer growth (127).

### Adipocytokines and Mechanisms Responsible for Bone Remodeling and the Formation of Osteolysis

Marrow adiposity has promoting effects on tumor-related osteolysis. Accelerated bone remodeling is one of the key factors associated with reactivation and growth of tumor cells colonized in the bone. Experimental treatment-induced osteoclasts formation and bone resorption, in turn, increase tumor cell growth and occurrences of bone metastases (128).

RANK signaling facilitates the differentiation of osteoclast progenitors via transcription factors like NF-κB and activator protein 1 (AP1) and by activating Jun N-terminal kinase (JNK), ERK1/2, and P38 MAPK, eventually stimulating nuclear factor of activated T-cells, cytoplasmic 1 (NFATc1), a master gene of osteoclastogenesis. Therefore, RANKL/RANK pathway is the predominant mediator of osteoclastogenesis, regulating bone resorption (129). After bone resorption, several growth factors stored in the bone matrix, such as TGF-β, platelet-derived growth factor (PDGF), IGF-1, and FGF, are released to promote cancer proliferation and establish a “vicious cycle” in osteolytic metastases (44).

#### Cytokines and Osteoclastogenesis

Cytokines, such as TNF-α, IL-1β, and IL-6, increase osteoclast activity by inducing the production of RANKL from osteoblasts and stromal cells, and decreasing OPG levels (44). For instance, the IL-6/IL-6R axis upregulates RANKL expression to induce osteoclast differentiation and bone resorption through JAK/STAT signaling (130). As described earlier, adipocytokines such as TNF-α, IL-1β, FGF-2, and CCL2 have also been found to be involved in the regulation of LOX expression in breast cancer (29, 39, 40). Interestingly, a transplantable breast cancer model shows that secreted LOX regulates bone homeostasis via osteoclastogenesis. LOX-mediated disruption of bone homeostasis is driven by NFATc1 directly and is independent of RANKL. High expression of LOX in tumors results in osteolytic lesion formation that could be inhibited by silencing or inhibition of LOX (38).

#### Adipokines and Osteoclastogenesis

Leptin and adiponectin show multiple functions in regulating bone homeostasis. Leptin can enhance the secretion of soluble intercellular adhesion molecule (sICAM)-1 by breast cancer cells to induce osteoclastogenesis and accelerate bone erosion (109). On the other hand, leptin acts on bone mesenchymal stem cells (MSCs) to promote their proliferation and differentiation of MSCs into osteoblasts (130).

In contrast, adiponectin inhibits osteoclastogenesis and resorption function by suppressing NF-κB and p38 signaling pathways, which is essential for osteoclast formation. Moreover, adiponectin blocks the formation of F-actin rings and attenuates osteoclast-mediated bone resorptive function (131). On the other hand, adiponectin activates the Wnt/β-catenin pathway in the MSCs to increase osteoblastic differentiation (132). Adiponectin also upregulates the expression of osteoblastic genes, such as osteocalcin, alkaline phosphatase, and Runx2 (133).

#### Novel Adipokines and Osteoclastogenesis

ANGPTL2 promotes osteoclastogenesis via upregulating NFATc1 expressions in macrophage colony-stimulating factor (M-CSF)-treated precursor cells (134). Chemerin receptor CMKLR1 is expressed by osteoclasts and mesenchymal stem cells (135). There is a paracrine role for chemerin in promoting osteoclasts differentiation through modulating intracellular calcium and NFATc1 (136). Chemerin also enhances mature osteoclast activity and bone resorption via extracellular signal-regulated kinase-5 (ERK5) phosphorylation. The activation of the ERK5 pathway boosts cathepsin K and matrix metalloproteinase-9 (MMP9) activity, a critical intracellular signaling cascade involved in the RANKL-induced osteoclastogenesis (135). LCN2 exerts a positive effect on bone resorption by increasing osteoclast maturation, through the enhancement of RANKL and IL-6 expression from osteoblasts (137, 138). Resistin shows dual functions in bone remodeling. On the one hand, resistin accelerates the proliferation of osteoblastic precursors (130). On the other hand, resistin facilitates osteoclasts differentiation via regulating protein kinase C (PKC) and PKA signaling pathways (139).

### Contribution of Adipocytokines to the Tumor Angiogenesis

Angiogenesis is necessary for the solid tumor to transport continuous oxygen and nutrient supply. It is also a crucial requirement of growth and progression for all subsets of breast cancer (140). Notably, the vasculature has an indispensable role in the formation of bone metastasis. Indeed, bone metastatic breast cancer cells prefer to colonize adjacent to the endothelial cells and even around the vessels (141). High vascularization supports cancer growth by providing nutrients and growth factors (7).

It is well-known that vascular endothelial growth factor (VEGF) is the crucial driver of angiogenesis. The function of VEGF is reinforced by the hypoxic condition presented in the marrow, hypoxia-inducible factor 1α (HIF-1α), and matrix metalloproteinases (MMPs) (142). Nonetheless, tumor cells can secrete angiogenic factors VEGF and promote the growth of capillaries into the tumor. Accumulating evidence suggests that adipocytokines can also regulate angiogenesis, thereby contributing to tumor progression (48). Adipocytes actively participate in angiogenic modulation through the secretion of adipocytokines, including leptin, IL-1β, IL-6, ANGPTL2, chemerin, FABP4, LCN2, resistin, and visfatin.

#### Leptin and Angiogenesis

In a paracrine manner, leptin is demonstrated to induce proliferation and migration of endothelial cells expressing Ob-R (143). Moreover, leptin stimulates blood-vessel growth in cooperation with VEGF. Leptin stimulation facilitates VEGF expression in breast cancer cells via HIF-1α and NF-κB (144). In breast cancer cell lines, treatment with leptin enhances cell proliferation, migration, and upregulation of VEGF and its receptor VEGFR-2 (145). This is highly dependent on the Notch, IL-1, and leptin cross-talk outcome (NILCO) in breast cancer. Thereby, NILCO is suggested as the integration of key signalings for leptin-induced tumor angiogenesis. In the short-term effect, leptin exerts pro-angiogenic actions via the direct transactivation of VEGFR-2 in endothelial cells. In the long term, this effect involves the upregulation of MMPs, integrins, and NILCO in breast cancer cells, which further promotes VEGF/VEGFR-2 expression (27, 146).

#### IL-1β, IL-6 and Angiogenesis

IL-1β stimulates the expression of VEGF and its receptor on endothelial cells. Also, IL-1β facilitates endothelial cell migration and tube formation via activating p38-MAPK (147). IL-6 influences HIF-1α and VEGF expression to regulate angiogenesis (142). In cancer cells, IL-6 upregulates VEGF expression by the JAK/STAT3 signaling (148). In a further study, chromatin immunoprecipitation indicates that the STAT3 activated by IL-6 binds to the VEGF promoter to stimulate VEGF production and accelerate tumor angiogenesis (149). Moreover, the effects of IL-6 on angiogenesis are involved in several other processes, such as enhancing endothelial progenitor cell migration, promoting vascular smooth muscle cell (VSMC) migration, and accelerating PDGF–mediated VSMC proliferation (150).

#### Novel Adipokines and Angiogenesis

Most ANGPTL proteins present angiogenic effects (52). The role of ANGPTL2 in angiogenesis is exhibited as a proangiogenic factor and exerts anti-apoptotic effects on endothelial cells (151). Existing data indicate that chemerin plays a role in the stimulation of endothelial cells proliferation, migration, and capillary tube formation (152). Further studies show that chemerin activated angiogenic effects are dependent on p42/44 MEK activation (153). FABP4 is a positive regulator of endothelial cell proliferation and angiogenesis, as a target of the VEGF/VEGFR2 pathway (153). LCN2 is reported to induce the production of HIF-1α and VEGF in breast cancer cells to stimulate angiogenesis, via the ERK signaling pathway (140). Visfatin facilitates endothelial proliferation and capillary tube formation in endothelial cells. This is mediated by increased production of VEGF and matrix metalloproteinases (MMP-2 and MMP-9) via MAPK/PI3K-Akt/VEGF signaling pathways (154). Visfatin also accelerates VSMC proliferation through nicotinamide mononucleotide-mediated activation of ERK 1/2 and p38 signaling pathways (155). In addition, visfatin reduces apoptosis in endothelial cells and induces maturation in human VSMC (153). Resistin upregulates VEGF expression in cancer cells to promote angiogenesis via PI3K/Akt signaling cascades (156).

Collectively, increased adipocytokines secretion from adipocytes, combined with the hypoxic microenvironment, establishes an ideal environment to drive angiogenesis via the upregulation of VEGF expression (142). This effect results in the development of new vasculature to support breast cancer metastatic growth.

## Conclusion and Prospects

As discussed above, BMAs have emerged as a crucial mediator of bone metastasis of breast cancer. Inhibiting BMAs is likely to lead to a novel therapeutic strategy for bone metastasis. BMAs are linked to osteoblasts by sharing the same progenitor, multipotent mesenchymal stromal cell. Adipocyte and osteoblast differentiation are closely related, and both types of cells share some common steps during their differentiation (12). This creates an inverse reciprocal relationship between osteoblastogenesis and adipogenesis. Some factors that promote one of the two processes usually inhibit the other (8). An approach is to regulate the balance between osteoblastogenesis and adipogenesis, thereby preventing an increase in marrow adiposity. Sclerostin is a Wnt signaling antagonist secreted by osteocytes, inhibiting osteoblastogenesis and new bone formation. Preclinical studies have shown a decreasing metastatic breast cancer burden in the mice bones with anti-sclerostin treatment (157). Interestingly, anti-sclerostin also reduces the volume of BMAs (158), implicating that the antitumor effect of sclerostin antibody may partly attribute to inhibiting BMAs (7). This treatment target follows the belief that “fat loss is bone gain” (14).

Another potential option is inhibiting the effects of adipocytokines secreted by BMAs. First, leptin peptide receptor antagonist is reported to suppress leptin-induced chemoresistances in breast cancer cells (159). This finding suggests leptin peptide receptor antagonist combined with chemotherapy improve chemosensitivity of breast cancer. Besides, IL-6 has been considered as a primary factor affecting the resistance of breast cancer to trastuzumab, a targeted therapeutic HER2 antibody. Blockade of IL-6 effect by an IL-6 antagonist, tocilizumab, reduces the breast cancer stem cell population, resulting in decreased cancer growth and metastasis in mice (160). Clinical trials are ongoing for investigating utilization of HER2 therapies in combination with IL-6 therapies to overcome drug resistance in HER2-positive breast cancer (54). Moreover, a clinical trial for triple-negative breast cancer is currently proceeding to test the checkpoint inhibitor PDR001 in combination with Canakinumab, an anti-IL-1β antibody (147). The results of this clinical trial will provide valuable information on the use of IL-1 antagonist in combined treatment. TNF-α neutralizing antibodies are also tested for cooperation with paclitaxel, a conventional chemotherapeutic agent in breast cancer. In mice, administration of TNF-α antibodies enhances the efficacy of paclitaxel treatment with respect to both breast cancer proliferation and lung metastasis (59). TNF-α neutralizing antibodies prove to be promising agents for their ability of suppressing metastasis as presented in animal models. When combined with eribulin, a chemotherapeutic microtubule inhibitor, a novel CXCL12/CXCR4 antagonist POL5551 reduces metastasis and prolongs survival in mice after resection of the primary breast cancer, compared with single-agent eribulin (161). However, more clinical trials are needed to assess these combined therapeutic approaches and their efficacy.

In conclusion, the bone marrow is highly enriched in adipocytes and it is the main metastatic site of breast cancer. Adipocytes are the most abundant components in the bone metastatic microenvironment that facilitate metastatic breast cancer cells in recruitment, invasion, survival, colonization, proliferation, angiogenesis, and immune modulation. BMAs are unique in their origin and location, and they serve as an endocrine organ via secreting adipokines, cytokines, chemokines, and growth factors. Most of these secreted adipocytokines are involved in pro-metastasis effects on breast cancer. Therefore, targeting BMAs combined with conventional treatment programs might present a promising therapeutic option for the bone metastasis of breast cancer. However, more studies should be performed to further uncover the complex interactions between BMAs and breast cancer cells in the bone microenvironment.

## Author Contributions

All authors listed have made a substantial, direct and intellectual contribution to the work, and approved it for publication.

## Conflict of Interest

The authors declare that the research was conducted in the absence of any commercial or financial relationships that could be construed as a potential conflict of interest.
